# Thermoelectric Properties of the Corbino Disk in Graphene

**DOI:** 10.3390/ma16124250

**Published:** 2023-06-08

**Authors:** Adam Rycerz, Katarzyna Rycerz, Piotr Witkowski

**Affiliations:** 1 Institute for Theoretical Physics, Jagiellonian University, Łojasiewicza 11, 30-348 Krakow, Poland; 2 Institute of Computer Science, AGH University of Science and Technology, al. Mickiewicza 30, 30-059 Krakow, Poland

**Keywords:** graphene, quantum Hall effect, thermopower, Landauer–Büttiker formalism

## Abstract

Thermopower and the Lorentz number for an edge-free (Corbino) graphene disk in the quantum Hall regime is calculated within the Landauer–Büttiker formalism. By varying the electrochemical potential, we find that amplitude of the Seebeck coefficient follows a modified Goldsmid–Sharp relation, with the energy gap defined by the interval between the zero and the first Landau levels in bulk graphene. An analogous relation for the Lorentz number is also determined. Thus, these thermoelectric properties are solely defined by the magnetic field, the temperature, the Fermi velocity in graphene, and fundamental constants including the electron charge, the Planck and Boltzmann constants, being independent of the geometric dimensions of the system. This suggests that the Corbino disk in graphene may operate as a thermoelectric thermometer, allowing to measure small temperature differences between two reservoirs, if the mean temperature magnetic field are known.

## 1. Introduction

Following the discovery of graphene [1,2,3], a two-dimensional form of carbon hosting ultra-relativistic effective quasiparticles [2,3], researchers were forced to re-examine numerous effects previously known from mesoscopic physics [4,5,6,7,8,9,10], prompting the development of the quantum Hall resistance standards [11,12,13], now considered the most important application to emerge from this field of research. Although the Hall bar setup is most commonly used (also in the study of artificial graphene analogues [14,15,16]), the edge-free Corbino geometry is often considered when discussing fundamental aspects of graphene  [6,17,18,19,20,21,22,23,24,25]. In such a geometry, magnetotransportion at high fields is unaffected by edge states, allowing one to probe the bulk transport properties [22,23,24]. Recently, sharp resonances in the longitudinal conductivity, associated with Landau levels, have been observed in graphene disks on hexagonal boron nitride [22].

In addition to conductivity measurements, thermoelectric phenomena including Seebeck and Nerst effects in graphene [26,27,28,29,30,31,32,33,34,35] and other two-dimensional systems [36,37,38,39] have been studied thoroughly, providing valuable insights into the details of the electronic structure of these materials. In particular, for systems with a wide bandgap $E_{g}$, the maximum absolute value of the Seebeck coefficient can be approximated by a Goldsmid–Sharp value [40,41]

(1) $${\left|S\right|}_{\max}\approx \frac{E_{g}}{2eT},\;\;\;\;\;\mathrm{for}\;\;\;E_{g}\gg k_{B}T,$$

with the absolute temperature *T*, the electron charge −e, and the Boltzmann constant $k_{B}$. (For more accurate approximations, see [31]).

The thermoelectric properties of graphene disks at zero (or low) magnetic fields have also been considered [42,43]. In the quantum Hall regime, thermoelectricity has been studied for GaAs/AlGaAs-based Corbino disks which host a two-dimensional gas of non-relativistic electrons [44,45,46,47]. However, analogous studies for graphene disks are currently missing.

**Figure 1:**
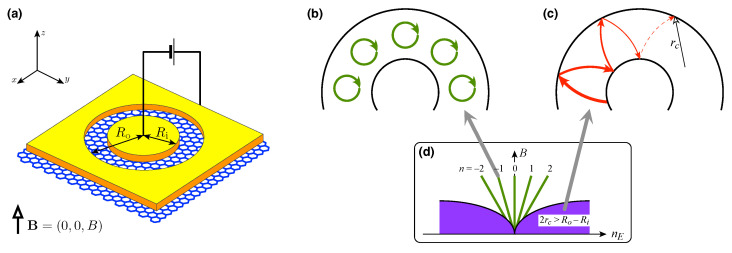
(**a**) The Corbino setup in graphene. Voltage source passes the current between the circular leads (yellow areas) via a disk-shaped sample with inner radii $R_{i}$ and outer radii $R_{o}$. The uniform magnetic field B=(0,0,B) is perpendicular to the sample. Additionally, the gate electrode (not shown) tunes the doping in the disk area. (**b**–**d**) Transport regimes for different fields and carrier concentrations $n_{E}$. At high fields, if doping is adjusted to a Landau level ($E=E_{n\mathrm{LL}}$, n=0,±1,…) resonance occurs (**b**). At low field but high doping (such that the cyclotron diameter $2r_{c}>R_{o}-R_{i}$), incoherent scattering along the classical trajectory governs the transport (**c**).

In this paper, we present numerical results on the Seebeck coefficient and Lorentz number (quantifying the ratio of thermal to electrical conductivity) for the ballistic disk in graphene (see Figure 1). The results show that although the deviations from Equation (1) are noticeable, the thermopower amplitude (determined by varying the doping at fixed temperature *T* and field *B*) can still be truncated by a closed-form function of the quantity $\Delta E_{\max}/\left(2eT\right)$, where $\Delta E_{\max}\propto \sqrt{B}$ is the maximum interval between the Landau levels (LLs), playing a role of the transport gap. A similar conclusion applies to the maximum Lorentz number. An unusual sequence of LLs in graphene leads to a relatively high thermoelectric response, expected for micrometre-sized disks at moderate fields B<0.5 T and temperatures of a few kelvins. The effect of smooth potential profiles is also discussed, introducing the electron-hole asymmetry of the transport properties [48,49].

The remaining parts of the paper are organized as follows. In Section 2 we present details of our numerical approach, which can be applied either in the idealized case where the electrostatic potential energy is a piecewise-constant function of the distance from the disk centre, or in the more general case of smooth potentials. Our numerical results for both cases are presented in Section 3. The conclusions are given in Section 4.

## 2. Model and Methods

### 2.1. Scattering of Dirac Fermions

Our analysis starts with the wave equation for massless Dirac fermions in graphene at energy *E* and uniform magnetic field *B*, which can be written as (for the *K* valley)

(2) $$\left[v_{F}\;\left(p+eA\right)\cdot \sigma +V\left(r\right)\right]\Psi =E\Psi ,$$

where $v_{F}=\sqrt{3}\;t_{0}a/\left(2\hbar \right)\approx 10^{6}\;$ m/s is the energy-independent Fermi velocity in graphene (with $t_{0}=2.7\;$ eV is the nearest-neighbour hopping integral and a=0.246 nm the lattice parameter), $p=(p_{x},p_{y})$ is the in-plane momentum operator with $p_{j}=-i\hbar \partial_{j}$, we choose the symmetric gauge $A=\frac{B}{2}\left(-y,x\right)$, and $\sigma =(\sigma_{x},\sigma_{y})$, where $\sigma_{j}$ are the Pauli matrices. The electrostatic potential energy V(r) depends only on the distance from the origin in polar coordinates (r,φ) and is given by

(3) $$V\left(r\right)=-V_{0}\times \left\{\begin{array}{cc} \;\frac{2^{m}{\left|r-R_{\mathrm{av}}\right|}^{m}}{|R_{o}-R_{i}{|}^{m}}\; & \mathrm{if}\;\;|r\;-\;R_{\mathrm{av}}|⩽\frac{R_{o}-R_{i}}{2},\\ \;1 & \mathrm{if}\;\;|r\;-\;R_{\mathrm{av}}|>\frac{R_{o}-R_{i}}{2}, \end{array}\right.$$

where $R_{\mathrm{av}}=\left(R_{i}+R_{o}\right)/2$ is defined with $R_{i}$ and $R_{o}$ being the inner and outer radii of the disk, respectively. We also note that the limit of $V_{0}\to \infty$ and m→∞ restores the familiar rectangular barrier of infinite height [18,19].

Because of the symmetry, the wave function can be written in the form

(4) $$\Psi_{j}\left(r,\varphi \right)=e^{i(j-1/2)\varphi }\left(\begin{array}{c} \chi_{a}\\ \chi_{b}e^{i\varphi } \end{array}\right),$$

where j=±1/2,±3/2,… is the total angular-momentum quantum number. In the leads, $r<R_{i}$ or $r>R_{o}$, the electrostatic potential energy is constant, $V\left(r\right)=-V_{0}$. In the case of electron doping, $E>-V_{0}$, the spinors ${\left(\chi_{a},\chi_{b}\right)}^{T}$ for the incoming (i.e., propagating from r=0) and outgoing (i.e., propagating from r=∞) waves are given, up to the normalization, by

(5) $$\chi_{j}^{\mathrm{in}}\;=\left(\begin{array}{c} H_{j-1/2}^{\left(2\right)}\left(kr\right)\\ iH_{j+1/2}^{\left(2\right)}\left(kr\right) \end{array}\right),\;\;\;\chi_{j}^{\mathrm{out}}\;=\left(\begin{array}{c} H_{j-1/2}^{\left(1\right)}\left(kr\right)\\ iH_{j+1/2}^{\left(1\right)}\left(kr\right) \end{array}\right),$$

where $H_{\nu }^{\left(1\right)}\left(\rho \right)$ ($H_{\nu }^{\left(2\right)}\left(\rho \right)$) is the Hankel function of the first (second) kind, $k=|E+V_{0}|/\left(\hbar v_{F}\right)$, and we have set B=0 in the leads [50]. (The wave functions for B≠0 are given explicitly in [17,19]). Full wave functions in the leads, for a given *j*, can be written as

(6) $$\begin{array}{ccc} \chi_{j}^{\left(i\right)} & =\chi_{j}^{\mathrm{in}}+r_{j}\chi_{j}^{\mathrm{out}},\; & r<R_{i}, \end{array}$$

(7) $$\begin{array}{ccc} \chi_{j}^{\left(o\right)} & =t_{j}\chi_{j}^{\mathrm{in}},\; & r>R_{o}, \end{array}$$

with the reflection (and transmission) amplitudes $r_{j}$ (and $t_{j}$).

For the disk area, $R_{i}<r<R_{o}$, we have B≠0 and the position-dependent V(r). Equation (2) gives the system of ordinary differential equations for spinor components

(8) $$\begin{array}{cc} \chi_{a}^{\prime } & =\left(\frac{j-1/2}{r}+\frac{eBr}{2\hbar }\right)\chi_{a}+i\;\frac{E-V(r)}{\hbar v_{F}}\chi_{b}, \end{array}$$

(9) $$\begin{array}{cc} \chi_{b}^{\prime } & =i\;\frac{E-V(r)}{\hbar v_{F}}\chi_{a}-\left(\frac{j+1/2}{r}+\frac{eBr}{2\hbar }\right)\chi_{b}, \end{array}$$

which has to be integrated numerically for all *j* s. To reduce round-off errors that occur in finite-precision pure mathematics due to exponentially growing (or decaying) spinor components, we have divided the interval $R_{i}\ldots R_{o}$ into *M* equally wide parts, bounded by $R_{i}^{\left(l\right)}<r<R_{o}^{\left(l\right)}$, with l=0,1,…,M−1, and

(10) $$R_{i}^{\left(l\right)}=R_{i}+l\;\frac{R_{o}-R_{i}}{M},\;\;\;\;\;R_{o}^{\left(l\right)}=R_{i}^{\left(l+1\right)}.$$

The resulting wave function for the *l*-th interval has the form

(11) $$\chi_{j}^{\left(l\right)}=A_{j}^{\left(l\right)}\chi_{j}^{(l),I}+B_{j}^{\left(l\right)}\chi_{j}^{(l),\mathrm{II}},$$

where $\chi_{j}^{(l),I}$ and $\chi_{j}^{(l),\mathrm{II}}$ denote the two linearly independent solutions obtained numerically by solving Equations (8) and (9) with two different initial conditions, ${\left.\chi_{j}^{(l),I}\right|}_{r=R_{i}^{\left(l\right)}}={\left(1,0\right)}^{T}$ and ${\left.\chi_{j}^{(l),\mathrm{II}}\right|}_{r=R_{i}^{\left(l\right)}}={\left(0,1\right)}^{T}$. $A_{j}^{\left(l\right)}$ and $B_{j}^{\left(l\right)}$ are arbitrary complex coefficients.

The matching conditions, namely

(12) $$\begin{array}{c} \chi_{j}^{\left(i\right)}\left(R_{i}\right)=\chi_{j}^{\left(0\right)}\left(R_{i}\right), \end{array}$$

(13) $$\begin{array}{cc} \chi_{j}^{\left(l\right)}\left(R_{o}^{\left(l\right)}\right) & =\chi_{j}^{\left(l+1\right)}\left(R_{i}^{\left(l+1\right)}\right),\;\;\;\;l=0,\ldots ,M-2, \end{array}$$

(14) $$\begin{array}{c} \chi_{j}^{\left(M-1\right)}\left(R_{o}\right)=\chi_{j}^{\left(o\right)}\left(R_{o}\right), \end{array}$$

are equivalent to the system of 2M+2 linear equations for the unknowns $A_{j}^{\left(0\right)}$, $B_{j}^{\left(0\right)}$,…, $A_{j}^{\left(M-1\right)}$, $B_{j}^{\left(M-1\right)}$, $r_{j}$, and $t_{j}$, which can be written as

$$\left[\begin{array}{ccccccc} -\chi_{j,a}^{\mathrm{out}}\left(R_{i}\right) & \chi_{j,a}^{(0),I}\left(R_{i}^{\left(0\right)}\right) & \chi_{j,a}^{(0),\mathrm{II}}\left(R_{i}^{\left(0\right)}\right) & & & & \\ -\chi_{j,b}^{\mathrm{out}}\left(R_{i}\right) & \chi_{j,b}^{(0),I}\left(R_{i}^{\left(0\right)}\right) & \chi_{j,b}^{(0),\mathrm{II}}\left(R_{i}^{\left(0\right)}\right) & & & & \\ 0 & \chi_{a}^{(0),I}\left(R_{o}^{\left(0\right)}\right) & \chi_{j,a}^{(0),\mathrm{II}}\left(R_{o}^{\left(0\right)}\right) & & & & \\ 0 & \chi_{b}^{(0),I}\left(R_{o}^{\left(0\right)}\right) & \chi_{j,b}^{(0),\mathrm{II}}\left(R_{o}^{\left(0\right)}\right) & \ddots & & & \\ & & & \ddots & \chi_{j,a}^{(\bar{M}),I}\left(R_{i}^{\left(\bar{M}\right)}\right) & \chi_{j,a}^{(\bar{M}),\mathrm{II}}\left(R_{i}^{\left(\bar{M}\right)}\right) & 0\\ & & & & \chi_{j,b}^{(\bar{M}),I}\left(R_{i}^{\left(\bar{M}\right)}\right) & \chi_{j,b}^{(\bar{M}),\mathrm{II}}\left(R_{i}^{\left(\bar{M}\right)}\right) & 0\\ & & & & \chi_{a}^{(\bar{M}),I}\left(R_{o}^{\left(\bar{M}\right)}\right) & \chi_{j,a}^{(\bar{M}),\mathrm{II}}\left(R_{o}^{\left(\bar{M}\right)}\right) & -\chi_{j,a}^{\mathrm{in}}\left(R_{o}\right)\\ & & & & \chi_{b}^{(\bar{M}),I}\left(R_{o}^{\left(\bar{M}\right)}\right) & \chi_{j,b}^{(\bar{M}),\mathrm{II}}\left(R_{o}^{\left(\bar{M}\right)}\right) & -\chi_{j,b}^{\mathrm{in}}\left(R_{o}\right) \end{array}\right]$$

(15) $$\times \;\left[\begin{array}{c} r_{j}\\ A_{j}^{\left(0\right)}\\ B_{j}^{\left(0\right)}\\ \vdots \\ A_{j}^{\left(M-1\right)}\\ B_{j}^{\left(M-1\right)}\\ t_{j} \end{array}\right]=\left[\begin{array}{c} \;\chi_{j,a}^{\mathrm{in}}\left(R_{i}\right)\;\\ \;\chi_{j,b}^{\mathrm{in}}\left(R_{i}\right)\;\\ 0\\ \vdots \\ 0 \end{array}\right],$$

where we have explicitly written the spinor components of the relevant wave functions appearing in Equations (12)–(14) and defined by $\bar{M}=M-1$.

Analogously, assuming the scattering from r=∞, we replace Equations (6) and (7) with

(16) $$\begin{array}{ccc} \chi_{j}^{\left(i\right)} & =t_{j}^{\prime }\chi_{j}^{\mathrm{out}},\; & r<R_{i}, \end{array}$$

(17) $$\begin{array}{ccc} \chi_{j}^{\left(o\right)} & =\chi_{j}^{\mathrm{out}}+r_{j}^{\prime }\chi_{j}^{\mathrm{in}},\; & r>R_{o}, \end{array}$$

and follow the consecutive steps mentioned above to obtain the linear system   

(18) $$A_{j}\times \left[\begin{array}{c} t_{j}^{\prime }\\ {A_{j}^{\left(0\right)}}^{\prime }\\ {B_{j}^{\left(0\right)}}^{\prime }\\ \vdots \\ {A_{j}^{\left(M-1\right)}}^{\prime }\\ {B_{j}^{\left(M-1\right)}}^{\prime }\\ r_{j}^{\prime } \end{array}\right]=\left[\begin{array}{c} 0\\ \vdots \\ 0\\ \;\chi_{j,a}^{\mathrm{out}}\left(R_{o}\right)\;\\ \;\chi_{j,b}^{\mathrm{out}}\left(R_{o}\right)\; \end{array}\right],$$

where $A_{j}$ is the main matrix as in Equation (15) with $r_{j}^{\prime }$ ($t_{j}^{\prime }$) being the reflection (transmission) amplitude for the scattering from the outer lead. Note that the elements of the $A_{j}$ matrix are unchanged; hence, Equations (8) and (9) only need to be numerically integrated once for a given *j*. The scattering matrix,

(19) $$S_{j}=\left[\begin{array}{cc} r & t^{\prime }\\ t & r^{\prime } \end{array}\right],$$

contains all the amplitudes mentioned above. Conservation of electrical charge implies unitarity of the $S_{j}$ matrix, $S_{j}S_{j}^{†}=S_{j}^{†}S_{j}=I$ (where I is the identity matrix). The deviation from unitarity due to numerical errors, i.e., $\max_{j}\left(|\epsilon_{j}|\right)$ with $\epsilon_{j}=\mathrm{Tr}\left(S_{j}^{†}S_{j}-I\right)$, provides a useful measure of the computational accuracy.

Since linear systems for different *j* s are decoupled, numerous software packages can be used to find their solutions up to machine precision. We use the double precision LAPACK routine zgesv, see [51]. The transmission probabilities were calculated as $T_{j}={\left|t_{j}\right|}^{2}$.

For heavily doped leads ($V_{0}\to \infty$) the wave functions given in Equation (5) simplify to

(20) $$\chi_{j}^{\left(\mathrm{in}\right)}=\frac{e^{iKr}}{\sqrt{r}}\left(\begin{array}{c} 1\\ 1 \end{array}\right),\;\;\;\;\chi_{j}^{\left(\mathrm{out}\right)}=\frac{e^{-iKr}}{\sqrt{r}}\left(\begin{array}{c} 1\\ -1 \end{array}\right),$$

with $K=|E+V_{0}|/\left(\hbar v_{F}\right)\to \infty$. In particular, for m→∞ and M=1, closed-form expressions for $T_{j}$ s were found, either for B=0 [18] or for B≠0 [19]. However, we found that the available implementations of hypergeometric functions that occur in these expressions lead to numerical stability problems when calculating thermoelectric properties in the quantum Hall regime. Therefore, a procedure described in this subsection was applied directly in the following numerical examples, with wave functions in the leads given by Equations (6) and (7) for m<+∞ (smooth potential barriers). For m→∞ (rectangular barrier), both the finite and infinite doping in the leads are studied for comparison.

The numerical integration of Equations (8) and (9) was performed for each M=20 interval using a standard fourth-order Runge–Kutta (RK4) algorithm. For $R_{o}=2R_{i}=1000$ nm and B<0.5 T, a spatial step of 0.5 pm was sufficient to reduce the unitarity error to $max\left(|\epsilon_{j}|\right)<10^{-8}$. The summation over the modes was stopped when $T_{j}<10^{-7}$. The Dormand–Prince method [52] was also implemented; however, no significant differences in the transmission probabilities were found compared to RK4.

### 2.2. Thermoelectric Characteristics

Within the Landauer–Büttiker formalism, the linear-response conductance [53,54] and other thermoelectric properties [55,56] can be calculated from the transmission-energy dependence

(21) $$T\left(E\right)=\sum_{j=-j_{\max}}^{j_{\max}}T_{j}\left(E\right),$$

where $j_{\max}=\left\lfloor KR_{i}\right\rfloor -\frac{1}{2}$ (for heavily doped leads, $j_{\max}\to \infty$), via dimensionless integrals

(22) $$L_{n}={\left(k_{B}T\right)}^{-n}\int dE\;T\left(E\right)\left(-\frac{\partial f_{\mathrm{FD}}}{\partial E}\right){\left(E-\mu \right)}^{n},$$

with $f_{\mathrm{FD}}\left(\mu ,T,E\right)=1/\left[\;\exp\left(\left(E\;-\;\mu \right)/k_{B}T\right)+1\;\right]$ the Fermi–Dirac distribution function and chemical potential μ. In particular,

(23) $$\begin{array}{cc} G & =\frac{g_{s}g_{v}e^{2}}{h}L_{0}\;\;\;\;\left(\mathrm{the}\;\mathrm{conductance}\right), \end{array}$$

(24) $$\begin{array}{cc} S & ={\left.\frac{dU}{dT}\right|}_{I=0}=\frac{k_{B}L_{1}}{eL_{0}}\;\;\;\;\left(\mathrm{the}\;\mathrm{Seebeck}\;\mathrm{coefficient}\right), \end{array}$$

(25) $$\begin{array}{cc} L & =\frac{K_{\mathrm{el}}}{TG}=\frac{k_{B}^{2}\left(L_{0}L_{2}\;-\;L_{1}^{2}\right)}{e^{2}L_{0}^{2}}\;\;\;\left(\mathrm{the}\;\mathrm{Lorentz}\;\mathrm{number}\right), \end{array}$$

where $g_{s}=g_{v}=2$ are the spin and valley degeneracies, ${dU/dT|}_{I=0}$ is the voltage derivative with respect to the temperature difference between the leads at zero electric current, and $K_{\mathrm{el}}$ is the electronic part of the thermal conductance.

For zero temperature, Equation (23) reduces to

(26) $$G\left(T\;\to \;0\right)=g_{0}T\left(E_{F}\right),$$

where the conductance quantum $g_{0}=4e^{2}/h$ and Fermi energy $E_{F}$ (=μ for T=0) are defined. In turn, the zero-temperature conductance provides a direct insight into the transmission-energy dependence.

For some specific T(E), integrals in Equations (24) and (25) can be calculated analytically. In particular, T(E)≈ const leads to S≈0 and $L\approx L_{0}=\left(\pi^{2}/3\right)\;k_{B}^{2}/e^{2}$, which defines the Wiedemann–Franz law for metals [57]. For gapless Dirac systems, the corresponding approximation is T(E)≈C|E|, with a constant C>0, for which both *S* and L can be expressed by a polylogarithm function of $\mu /k_{B}T$[30], with a universal (C-independent) maxima $S_{\max}≃1.0023\;k_{B}/e$ and $L_{\max}≃2.3721\;L_{0}$. The latter value was first reported in the context of *d*-wave systems [58], before being found again for Dirac materials [59,60,61].

In the presence of a transport gap, one can consider a simplified model for T(E), given by

(27) $$T_{\mathrm{model}}\left(E\right)=A\delta \left(E\right)+B\delta \left(E-\Delta E\right),$$

where A>0 and B>0 are constants, and δ(x) is the Dirac delta function. Generalizing the derivations presented in [31,42] to the asymmetric case (A≠B), one easily finds

(28) $$\begin{array}{cc} \frac{S_{\max}\;-\;S_{\min}}{2} & ≃\frac{k_{B}}{e}\;\left[\sqrt{u(u\;-\;1)}-\ln\left(\sqrt{u}+\sqrt{u\;-\;1}\right)\right], \end{array}$$

(29) $$\begin{array}{cc} L_{\max} & ≃{\left(\frac{k_{B}}{e}\right)}^{2}u^{2}={\left(\frac{\Delta E}{2eT}\right)}^{2}, \end{array}$$

where $u=\Delta E/(2k_{B}T)$ and the asymptotic equalities correspond to u≫1. The remaining symbols in Equation (28) are the maximum ($S_{\max}$) and minimum ($S_{\min}$) Seebeck coefficient in the interval of 0<μ<ΔE. Note that the right-hand sides in Equations (28) and (29) only depend on *u* and fundamental constants.

In a case where more δ-shaped peaks appear in the transmission spectrum T(E), as might be expected for the quantum Hall regime, the approximations given by Equations (28) and (29) are also valid, provided a gap is identified with the maximum interval between the peaks ($\Delta E=\Delta E_{\max}$). The monotonicity of the right-hand sides of Equations (28) and (29) guarantees that the resulting approximations, for $\Delta E=\Delta E_{\max}$, correspond to the global maxima of the relevant quantities (i.e., the thermopower amplitude and Lorentz number) as functions of μ.

## 3. Results and Discussion

### 3.1. Zero-Temperature Conductance

Before discussing the thermoelectric properties, we present zero-temperature conductance spectra, related to the transmission-energy dependence via Equation (26), thus representing the input data for the Seebeck coefficient and Lorentz number calculations. For the rectangular barrier of infinite height, $m,V_{0}\to \infty$ in Equation (3), the spectra are particle-hole symmetric, and it is sufficient to consider μ⩾0.

**Figure 2:**
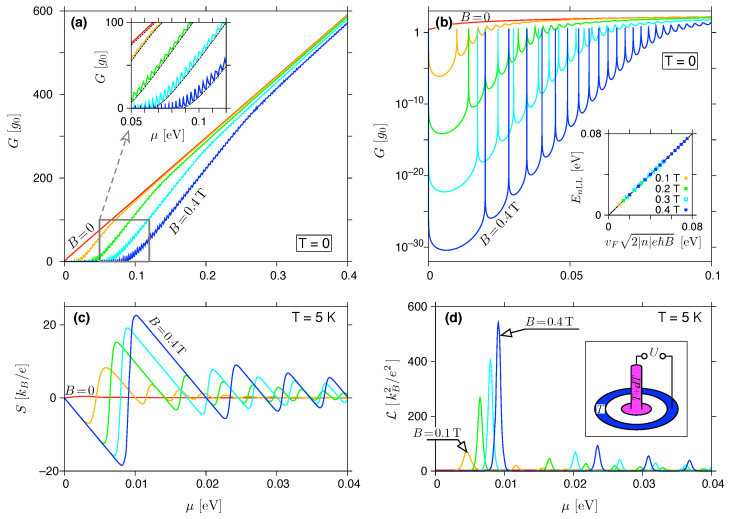
(**a**,**b**) Zero-temperature conductance, (**c**) the Seebeck coefficient and (**d**) the Lorentz number, both for T=5 K, for the system of Figure 1 with $R_{o}=2R_{i}=1000\;$ nm and the rectangular potential barrier ($V_{0},m\to \infty$; see Equation (3)) displayed as functions of the chemical potential. The magnetic field is varied from B=0 (red solid lines in all plots) to B=0.4 T (blue solid lines) with steps of 0.1 T. Inset in (**a**) is zoomed-in, with black dashed lines depicting the incoherent conductance (see Appendix A). (**b**) shows the same data as (**a**), but using a semi-logarithmic scale, with the inset presenting positions of the actual transmission maxima for B>0 ($E_{n\mathrm{LL}}$) versus the values for bulk graphene (see Equation (30)). A setup for the thermoelectric measurements is also depicted (see inset in (**d**)).

In Figure 2a,b we display the disk conductance as a function of $\mu =E_{F}$ for T=0. Resonances via the Landau levels are centred near energies very close to the corresponding values for bulk graphene,

(30) $$E_{n\mathrm{LL}}\approx E_{n\mathrm{LL}}^{\mathrm{bulk}}=\mathrm{sgn}\left(n\right)v_{F}\sqrt{2|n|e\hbar B},$$

where *n* is an integer (without generality loss, we hereinafter suppose B>0) clearly visible starting from a moderate value of B=0.1 T. More generally, one can expect the resonances to be visible up to the *n*-th resonance if

(31) $$E_{n\mathrm{LL}}≲E_{c,1}=\frac{v_{F}eB}{2}\left(R_{o}-R_{i}\right),$$

where $E_{c,1}$ denotes the threshold energy, below which the cyclotron diameter $2r_{c}=2\left|E_{F}\right|/\left(v_{F}eB\right)<R_{o}-R_{i}$ and the incoherent transmission vanishes (Appendix A). For instance, for $R_{o}=2R_{i}=1000\;$ nm discussed throughout the paper, Equation (31) gives

(32) |n|≲47.47×B[T],

coinciding with the number of well-separated maxima depicted in semi-logarithmic scale in Figure 2b.

It is also visible, for $|E_{F}|>E_{c,1}$, that the actual *G* grows rapidly with increasing $|E_{F}|$, closely following the prediction for incoherent transport (see Figure 2a and the inset). This observation leads to the question of whether $E_{1\mathrm{LL}}$, or rather $2E_{c,1}$, defines the relevant transport gap to be substituted into Equations (28) and (29)?

This problem is solved via the numerical analysis of *S* and L presented next.

### 3.2. Thermopower and the Lorentz Number

The Seebeck coefficient and Lorentz number, calculated from Equations (24) and (25) for T=5 K, are shown in Figure 2c,d as functions of the chemical potential. In addition, the particle-hole symmetry of T(E) guarantees that S(μ) is odd and L(μ) is even on μ→−μ, and it is sufficient to consider μ⩾0.

In the quantum Hall regime, i.e., for $|\mu |<E_{c,1}$, see Equation (31), the T(E) function consists of narrow peaks, each centred at $E_{n\mathrm{LL}}$ (see Section 3.1). In such a case, reliable numerical estimations of the integrals $L_{0}$, $L_{1}$, and $L_{2}$ (see Equation (22)) requires sufficiently dense sampling of T(E) near $E\approx E_{n\mathrm{LL}}$.

The analytic structure of Equations (24) and (25) results in the following features visible in Figure 2c,d. First, each of the consecutive intervals, i.e., $E_{0\mathrm{LL}}<E<E_{1\mathrm{LL}}$, $E_{1\mathrm{LL}}<E<E_{2\mathrm{LL}}$, etc., contains a local minimum and maximum of *S*, surrounding an odd zero of *S* (even zeros occur for the resonances at $\mu \approx E_{n\mathrm{LL}}$), corresponding to a local maximum of L. Second, the global extrema (corresponding to $S_{\min}$, $S_{\max}$, or $L_{\max}$) are all in the first interval, $E_{0\mathrm{LL}}<E<E_{1\mathrm{LL}}$, characterized by the maximum width ($\Delta E_{\max}\equiv E_{1\mathrm{LL}}-E_{0\mathrm{LL}}=E_{1\mathrm{LL}}$).

**Figure 3:**
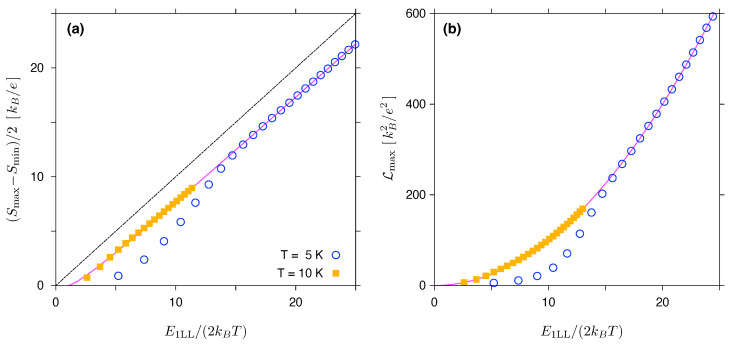
(**a**) Maximum amplitude of the Seebeck coefficient and (**b**) maximum Lorentz number for same system as in Figure 2 at T=5 K (open symbols) and T=10 K (closed symbols) as functions of the maximum interval between the Landau levels of $\Delta E_{\max}\equiv E_{1\mathrm{LL}}=\left|E_{0\mathrm{LL}}-E_{\pm 1\mathrm{LL}}\right|$. Solid lines depict the asymptotic expressions given in Equations (28) and (29). Dashed line in (**a**) corresponds to the Goldsmid–Sharp relation, see Equation (1), with $E_{g}=E_{1\mathrm{LL}}$.

In Figure 3, the values of $(S_{\max}-S_{\min})/2$ and $L_{\max}$ are plotted against the dimensionless variable $E_{1\mathrm{LL}}/\left(2k_{B}T\right)$, for the two values of T=5 K and 10 K. Results of the numerical integration and subsequent optimization with respect to the chemical potential μ (data points) closely follow the approximations given in Equations (28) and (29) (solid lines), starting from $E_{1\mathrm{LL}}/\left(2k_{B}T\right)≳10$. The Goldsmid–Sharp formula (dashed line) produces a noticeable offset when compared to the Landauer–Büttiker results; however, the formula can still be used as a less accurate approximation for $(S_{\max}-S_{\min})/2$ for large $E_{1\mathrm{LL}}/\left(2k_{B}T\right)$.

These results support our conjecture that the model T(E), given by Equation (27), is able to reproduce the basic thermoelectric properties of a graphene disk in the quantum Hall regime. Although, at first glance it may seem surprising that the model T(E) with $\Delta E=E_{1\mathrm{LL}}$ reproduces the actual numerical results, the energy scale of $2E_{c,1}\gg E_{1\mathrm{LL}}$ (see Equation (31)) seems to be irrelevant. However, for thermal excitation energies $k_{B}T\ll E_{1\mathrm{LL}}\ll 2E_{c,1}$, the detailed behaviour of the actual T(E) (see Equation (21)) for $E-E_{0\mathrm{LL}}=E≲-k_{B}T$ or $E-E_{1\mathrm{LL}}≳k_{B}T$ does not affect the integrals $L_{0}$, $L_{1}$ and $L_{2}$ (note that the full width at half maximum for $-\partial f_{\mathrm{FD}}/\partial E$ in Equation (22) is $\approx \;3.53\;k_{B}T\;$) when $0<\mu <E_{1\mathrm{LL}}$. For this reason, a model with $\Delta E=E_{1\mathrm{LL}}$ captures the essential features of the actual T(E), while focussing on the thermoelectric properties considered here.

### 3.3. Smooth Potential Barriers

**Figure 4:**
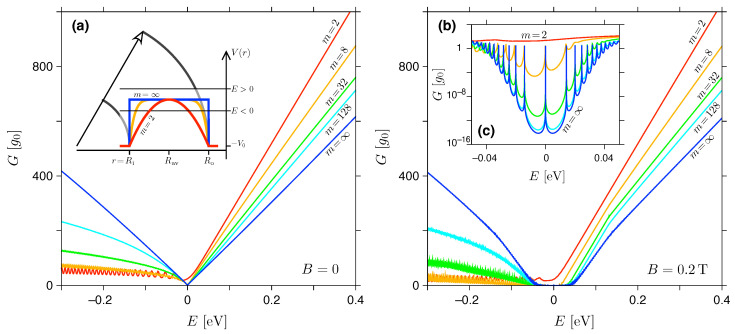
Zero-temperature conductance at (**a**) B=0 and (**b**) B=0.2 T versus the Fermi energy. The disk radii are the same as in Figure 2, the barrier height (see Equation (3)) is fixed at $V_{0}=t_{0}/2=1.35\;$ eV, and the parameter *m* is specified for each line. Inset in (**a**) shows the selected potential profiles. (**c**) Zoom-in, for low energies, with the same datasets as in (**b**) displayed on a semi-logarithmic scale.

For the sake of completeness, in this section we revisit the effects of smooth potential barriers, considered earlier for the zero magnetic field [49]. The electrostatic potential energy is given by Equation (3), where the barrier height is fixed at $V_{0}=t_{0}/2=1.35\;$ eV (for selected profiles for m=2, 8, and *∞*, see Figure 4) and the radii $R_{o}=2R_{i}=1000$ nm again.

For a finite barrier height, the particle-hole symmetry of the conductance spectrum G(E) is absent, even for the rectangular barrier (m=∞). However, for sufficiently low energies $\left|E\right|\ll V_{0}$ and large *m*, the Fermi wavelength $\lambda_{F}=hv_{F}/\left|E\right|$ becomes longer than the characteristic length scale of a potential jump, i.e., $\Delta r=(L-L_{\mathrm{eff}})/2$ where the sample length is $L=R_{o}-R_{i}=500\;$ nm, and

(33) $$L_{\mathrm{eff}}^{\left(m\right)}=L{\left(\frac{\hbar v_{F}}{LV_{0}}\right)}^{1/m}$$

is the so-called diffusive length defined via $V\left(R_{\mathrm{av}}\pm L_{\mathrm{eff}}/2\right)=-\hbar v_{F}/L$ (see [48]). The value of $\hbar v_{F}/L$ can be attributed to the energy uncertainty corresponding to a typical time of flight $∼L/v_{F}$ (up to the order of magnitude). We further note that $L_{\mathrm{eff}}^{\left(m\right)}\to L$ for m→∞. If $\lambda_{F}\gg \Delta r$, the potential profile can be considered approximately flat, and the approximate symmetry upon E→−E can be observed for the corresponding zero-field spectra shown in Figure 4a.

For B=0.2 T, see Figure 4b,c, the approximate symmetry is also visible. In addition, it is worth noting that for $\lambda_{F}\gg \Delta r$ the lowest LLs are well pronounced, and their positions are almost unaffected compared to the infinite-barrier case (see previous subsection, Figure 2b).

In both cases, i.e., for B=0 and B=0.2 T, the presence of two circular p-n junctions for E<0 leads to a suppressed conductance compared to E>0, with well-pronounced conductance oscillations due to quasi-bounded states (especially for smaller *m*). Due to this asymmetry, the global conductance minimum $G_{\min}$ in the quantum Hall regime is typically reached in the energy interval of $-E_{1\mathrm{LL}}<E<0$, with the exception of the parabolic profile (m=2), for which resonances with LLs are obliterated.

The consequences of the above-mentioned features of G(E) for the thermoelectric properties are discussed next.

**Figure 5:**
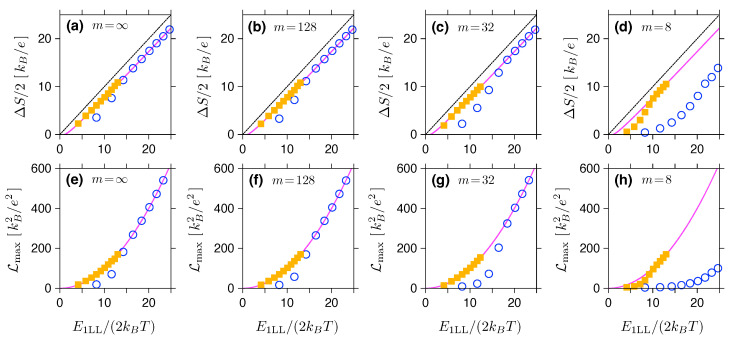
(**a**–**d**) Maximum amplitude of the Seebeck coefficient $\Delta S=(S_{\max}-S_{\min})/2$ and (**e**–**h**) Lorentz number for the same system in Figure 4 and selected values of the exponent *m* defining the potential profile (see Equation (3)) displayed as the bulk Landau-level energy $E_{1\mathrm{LL}}$ obtained from Equation (30) with n=1. The line/colour encoding is same as in Figure 3.

In Figure 5 we show pairs of plots analogous to those shown in Figure 3, i.e., the thermopower amplitude $\Delta S=(S_{\max}-S_{\min})/2$, and the maximum Lorentz number $L_{\max}$, both displayed as functions of the dimensionless quantity $E_{1\mathrm{LL}}/\left(2k_{B}T\right)$. This time the step height is finite and four values of the exponent *m* (specified for each panel) are chosen. For sufficiently large $E_{1\mathrm{LL}}/\left(2k_{B}T\right)$, the approximations of Equations (28) and (29) (solid lines) are closely followed by the actual data points for smooth potentials (m<+∞). We further note that the agreement is generally better for T=5 K (solid symbols) than for T=10 K (open symbols).

**Figure 6:**
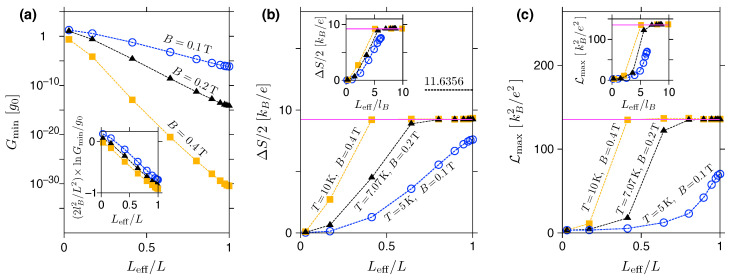
(**a**–**c**) Thermoelectric characteristics of the disk with smooth potential barriers in the quantum Hall regime displayed versus $L_{\mathrm{eff}}$ given by Equation (33). (**a**) The minimum zero-temperature conductance reached for $-E_{1\mathrm{LL}}<E<0$, with the inset depicting the scaling according to $G_{\min}\propto \exp\left(-\frac{1}{2}L_{\mathrm{eff}}^{2}/l_{B}^{2}\right)$ with $l_{B}=\sqrt{\hbar /eB}$ for the magnetic length. (**b**) The maximum thermopower amplitude. (**c**) The maximum Lorentz number. Insets in (**b**,**c**) show the same data as functions of the rescaled length $L_{\mathrm{eff}}/l_{B}$. The magnetic field is varied between the datasets (see data points, dashed lines are only a guide). Additionally, in (**b**,**c**) the temperature is varied to keep the constant $E_{1\mathrm{LL}}/\left(2k_{B}T\right)≃11.6356$. Solid horizontal lines in (**b**,**c**) mark the values from Equations (28) and (29).

The effect of $L_{\mathrm{eff}}/L<1$ corresponding to m<+∞, see Equation (33), and quantifying the potential smoothness is further illustrated in Figure 6. Here we show the selected thermoelectric properties, $G_{\min}$, ΔS, and $L_{\max}$, as functions of $L_{\mathrm{eff}}/L$. Each dataset corresponds to a fixed magnetic field (B=0.1 T, 0.2 T, or 0.4 T), while the exponent *m* is varied from m=2 to 512, with an additional data point for the rectangular barrier (m=∞) placed at $L_{\mathrm{eff}}/L=1$. For finite-temperature characteristics, ΔS and $L_{\max}$, we set T=5 K for B=0.1 T; otherwise, *T* is chosen to keep the constant $E_{1\mathrm{LL}}/\left(2k_{B}T\right)$ ratio (a quantity determining the approximate values ΔS and $L_{\max}$ via Equations (28) and (29)).

Remarkably, the datasets for different *B* values reveal common behaviours upon proper rescaling (see the three insets in Figure 6a–c). In the first plot, it is easy to see that the conductance away from the resonances with LLs behaves approximately as $G_{\min}\propto \exp\left(-\frac{1}{2}L_{\mathrm{eff}}^{2}/l_{B}^{2}\right)$. In the next two plots, the datasets for the finite-temperature characteristics (ΔS and $L_{\max}$) come much closer to each other if plotted as functions of $L_{\mathrm{eff}}/l_{B}$ (where $l_{B}=\sqrt{\hbar /eB}≃25.656\;$ nm ×(B[T${])}^{-1/2}$ is the magnetic length) than if simply plotted as functions of $L_{\mathrm{eff}}$.

In addition, the behaviour of $G_{\min}$ further validates the numerical stability of the approach presented in Section 2. Namely, the value of $G_{\min}/g_{0}∼10^{-30}$ corresponds to the transmission amplitude $|t_{j}|∼10^{-15}$, which coincides with a typical round off error in double-precision mathematics. This is also why we have limited our discussion to B⩽0.4 T (or, equivalently, $L/l_{B}⩽12.33$ for L=500 nm). For higher *B*, one must use numerical tools employing multiple precision strategies [62]. In such a case, a significant slowdown of the computations is expected.

From a physical point of view, the considered values of *B* lead to the Zeeman splitting of $\Delta E_{Z}=g\mu_{B}B\approx 1.16\cdot 10^{-4}\;$ eV ×B[T$]\ll E_{1\mathrm{LL}}$, with g≃2 and $\mu_{B}=e\hbar /\left(2m_{e}\right)$ the Bohr magneton. Throughout this paper the Zeeman term is therefore neglected.

## 4. Conclusions

We have investigated the selected thermoelectric properties of graphene-based Corbino disks in the presence of an external magnetic field. An efficient numerical scheme was proposed allowing for the determination of these properties through mode matching for the Dirac equation, up to the magnetic fields that drive the system into the quantum Hall regime, using only a standard double precision method.

Our results show that both the thermopower amplitude and maximum Lorentz number are determined by the energy interval separating the n=0 and n=±1 LLs divided by the absolute temperature and fundamental constants. The ratio of the disk radii and the detailed shape of the electrostatic potential profile are irrelevant. Approximate expressions for the two above-mentioned thermoelectric characteristics can be derived by assuming the transmission-energy dependence to be in the form of two Dirac-delta peaks, centred around the n=0 and n=1 (or n=−1) LL energies. In particular, the expression describing the thermopower amplitude can be regarded as a modified version of the well-known Goldsmid–Sharp relation for semiconductors. It appears that a disk-shaped graphene sample, coupled to the two reservoirs in local thermal equilibrium, can act as a thermometer measuring the small temperature difference between the reservoirs (provided that the applied field and average temperature are known).

Our analysis was carried out within the Landauer–Büttiker formalism for non-interacting quasi-particles. This implies that the fractional quantum Hall effect (FQHE) is outside the scope of this work. Although the transmission resonances with FQHE states have been observed in ultra-clean graphene samples [22], existing thermoelectric measurements for GaAs/AlGaAs disks [45,47] indicate the presence of integer QHE states only. For this reason, a theoretical study of the thermoelectric signatures of integer QHE states had to be completed as a first step. Undoubtedly, generalizing the approach to include FQHE states would be a promising direction for future studies.

## Figures and Tables

**Figure A1:**
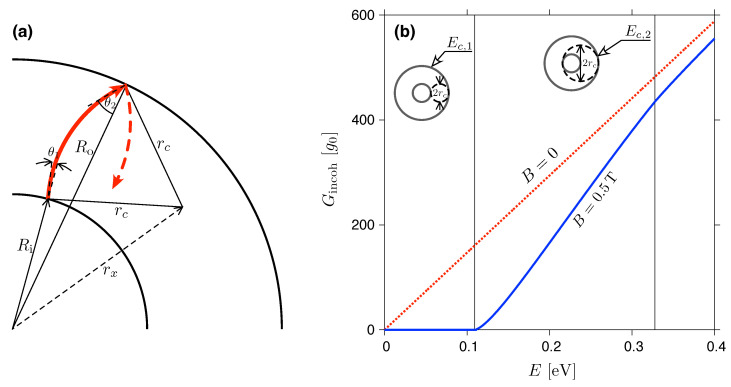
(**a**) Propagation between subsequent scatterings on interfaces at $r=R_{i}$ and $r=R_{o}$ (with incident angles $\theta_{1}$ and $\theta_{2}$, respectively) in a uniform magnetic field, defining the cyclotron orbit centred at $r=r_{x}$ with its radii $r_{c}$. (**b**) Incoherent conductance calculated from Equation (A1) for B=0 (dashed line) and B=0.5 T (solid line). The disk radii are $R_{o}=2R_{i}=1000\;$ nm; a rectangular potential barrier is considered. Characteristic Fermi energies $E_{c,1}$ and $E_{c,2}$ marked with vertical lines correspond to $r_{c}=\left(R_{o}-R_{i}\right)/2$ and $r_{c}=\left(R_{i}+R_{o}\right)/2$, respectively.

## Data Availability

Original data are available from the authors upon resonable request.

## Appendix A. Incoherent Transport at the Magnetic Field

Incoherent conductance ($G_{\mathrm{incoh}}$) is calculated below by adapting the method presented in [49] for a uniform magnetic field.

Due to disk symmetry, the incident angles, $\theta_{1}$ for the interface at $r=R_{i}$ and $\theta_{2}$ for the interface at $r=R_{o}$ (see Figure A1) remain constant (up to a sign) during multiple scattering between the two interfaces. In turn, for a multi-mode regime, Equation (26) can be approximated by averaging over the modes, leading to

(A1) $$G_{\mathrm{incoh}}=2g_{0}k_{F}R_{i}\langle T_{12}\rangle ,$$

where

(A2) $$\langle T_{12}\rangle =\frac{1}{2}\int_{u_{c}}^{1}du_{1}\frac{T_{1}T_{2}}{T_{1}+T_{2}-T_{1}T_{2}},$$

with

(A3) $$T_{j}=\frac{2\sqrt{1-u_{j}^{2}}}{1+\sqrt{1-u_{j}^{2}}},\;\;\;\;j=1,2,$$

being the transmission probabilities for a potential step of infinite height. The parameter $u_{1}=\sin\theta_{1}$ is further regarded as an independent variable. $u_{c}$ in Equation (A2) denotes the minimal value of $u_{1}$ above which the corresponding $u_{2}=\sin\theta_{2}$ satisfies $|u_{2}|\;⩽\;1$.

Assuming a constant electrostatic potential energy in the disk area, the trajectory between subsequent scatterings forms an arc, with radii $r_{c}=\left|E\right|/\left(v_{F}eB\right)$ (the cyclotron radius for a massless Dirac particle at a field B>0), centred at distance $r_{x}$ from the origin. Solving the two triangles with common edge $r_{x}$ (dashed line) and the opposite vertices in two scattering points, one easily finds

(A4) $$r_{x}^{2}=R_{i}^{2}+r_{c}^{2}+2R_{i}r_{c}\sin\theta_{1}$$

(for the triangle containing a scattering point at $r=R_{i}$), and

(A5) $$r_{x}^{2}=R_{o}^{2}+r_{c}^{2}-2R_{o}r_{c}\sin\theta_{2}$$

(for the triangle containing a scattering point at $r=R_{o}$), leading to

(A6) $$u_{2}=\sin\theta_{2}=\frac{R_{o}^{2}-R_{i}^{2}-2R_{i}r_{c}u_{1}}{2R_{o}r_{c}}.$$

Therefore, the value of $u_{c}$ in Equation (A2) is given by

(A7) $$u_{c}=\left\{\begin{array}{cc} -1, & \mathrm{if}\;\;r_{c}⩾\frac{R_{i}+R_{o}}{2}\\ \frac{R_{o}^{2}-R_{i}^{2}}{2R_{i}r_{c}}-\frac{R_{o}}{R_{i}},\; & \mathrm{if}\;\;\frac{R_{i}+R_{o}}{2}>r_{c}⩾\frac{R_{o}-R_{i}}{2}\\ 1, & \mathrm{if}\;\;r_{c}<\frac{R_{o}-R_{i}}{2} \end{array}\right..$$

In a zero-field limit, we have $r_{c}\to \infty$, leading to $u_{c}=-1$ and $u_{2}\to -\left(R_{i}/R_{o}\right)u_{1}$. In such a limit, the integral in Equation (A2) can be calculated analytically, leading to

(A8) $$G_{\mathrm{incoh}}\left(B\to 0\right)=2g_{0}k_{F}R_{i}\frac{\left(2a+\frac{1}{a}\right)\arcsin a+3\sqrt{1-a^{2}}-\frac{\pi }{2}\left(a^{2}+2\right)}{1-a^{2}},\;\;\;\;\mathrm{with}\;\;a=R_{i}/R_{o}.$$

The above reproduces a zero-field result as reported in [49]. For B>0, the integration needs to be performed numerically.

From a geometric perspective, the limiting values of $r_{c}$ in Equation (A7), i.e., $r_{c,1}=\left(R_{o}-R_{i}\right)/2$ and $r_{c,2}=\left(R_{i}+R_{o}\right)/2$, indicate three distinct situations: (i) none of the circular trajectories originating from $r=R_{i}$ can reach $r=R_{o}$ (the $r_{c}<r_{c,1}$ case); (ii) trajectories with some incident angles $\theta_{1}$ may reach the second interface, but some cannot (the $r_{c,2}>r_{c}⩾r_{c,1}$ case); and (iii) all the trajectories originating from $r=R_{i}$ reach $r=R_{o}$ (the $r_{c}⩾r_{c,1}$ case).

Furthermore, in Figure A1, we display $G_{\mathrm{incoh}}$, calculated numerically from Equation (A1) for B=0 and B=0.5 T, with the remaining system parameters considered throughout the paper. The Fermi energies $E_{c,\alpha }=v_{F}eBr_{c,\alpha }$, α=1,2, for B=0.5 T, are marked with vertical lines. It can be shown that for $E\approx E_{c,1}$, the incoherent conductance behaves as

(A9) $$G_{\mathrm{incoh}}\left(E\right)\propto \Theta \left(E-E_{c,1}\right){\left|E-E_{c,1}\right|}^{3/2},$$

with Θ(x) being the Heaviside step function.

Remarkably (see the main text), $G_{\mathrm{incoh}}$ closely follows the actual *G* calculated via the numerical mode matching, but the thermoelectric characteristics are ruled by the LLs, with their energies being unrelated to the value of $E_{c,1}$.
